# Sex differences in the associations between vagal reactivity and oppositional defiant disorder symptoms

**DOI:** 10.1111/jcpp.12750

**Published:** 2017-06-02

**Authors:** Pablo Vidal‐Ribas, Andrew Pickles, Florin Tibu, Helen Sharp, Jonathan Hill

**Affiliations:** ^1^ Department of Child and Adolescent Psychiatry Institute of Psychiatry, Psychology and Neuroscience King's College London London UK; ^2^ Department of Biostatistics and Health Informatics Institute of Psychiatry, Psychology and Neuroscience King's College London London UK; ^3^ Institute of Child Development Bucharest Early Intervention Project Lab Bucharest Romania; ^4^ Institute of Psychology, Health and Society University of Liverpool Liverpool UK; ^5^ School of Psychology and Clinical Language Sciences University of Reading Reading UK

**Keywords:** Vagal reactivity, oppositional defiant disorder, irritability, headstrong, sex differences

## Abstract

**Background:**

Vagal reactivity to stress in children has been associated with future psychiatric outcomes. However, results have been mixed possibly because these effects are in opposite direction in boys and girls. These sex differences are relevant in the context of development of psychopathology, whereby the rates of psychiatric disorders differ by sex. In this study, we aimed to examine the association between vagal reactivity, assessed as a reduction in respiratory sinus arrhythmia (RSA) in response to a challenge, and the development of future oppositional defiant disorder (ODD) symptoms in boys and girls. In addition, we examine the specific associations with ODD symptom dimensions, named irritability and headstrong. We hypothesized that increased vagal reactivity was associated with increased ODD symptoms in girls and a reduction in ODD symptoms in boys.

**Methods:**

Participants were members of the Wirral Child Health and Development Study, a prospective epidemiological longitudinal study of 1,233 first‐time mothers recruited at 20 weeks’ gestation. RSA during four nonstressful and one stressful (still‐face) procedures was assessed when children were aged 29 weeks in a sample stratified by adversity (*n* = 270). Maternal reports of ODD symptoms were collected when children were 2.5 years old (*n* = 253), 3.5 years old (*n* = 826), and 5 years old (*n* = 770). Structural equation modeling (*SEM*) was employed to test our hypotheses.

**Results:**

There was a significant sex difference in the prediction of ODD symptoms due to the opposite directionality in which increasing vagal reactivity was associated with an increase in ODD symptoms in girls and a reduction of ODD symptoms in boys. This Sex by Vagal reactivity interaction was common for both ODD dimensions, with no sex by dimension‐specific associations.

**Conclusions:**

Physiological reactivity to a stressful situation predicts differently ODD symptoms in boys and girls very early in life, with no difference across irritability and headstrong components. Findings are discussed in the context of the several mechanisms involved on the later development of distinct psychiatric disorders in boys and girls.

## Introduction

It has been suggested that vagal reactivity, otherwise known as vagal withdrawal, plays a role in emotional regulation (Porges, 2007), which is impaired in many children with oppositional defiant disorder (ODD) (Cavanagh, Quinn, Duncan, Graham, & Balbuena, 2017). However, results are inconsistent (Graziano & Derefinko, 2013). One possible reason is that there may be opposite effects of vagal reactivity in males and females (Eisenberg et al., 1995). In this study, we examine whether there are sex differences in associations between vagal reactivity in infancy and ODD symptoms up to the age of 5 years.

Porges's *Polyvagal Theory* (Porges, 2007) asserts that engagement with the environment is physiologically mediated by the ventral vagal complex. Pathways originating in the nucleus ambiguous, which regulates vagal influence on the heart, are linked to those that regulate muscles controlling facial expressions and vocalizing. It is hypothesized that the adaptive response to stress entails a reduction in vagal tone, which mobilizes physiological and psychological resources to underpin effective action without activating flight‐fight associated with the sympathetic nervous system. This hypothesis has been supported by several studies that found associations between greater vagal reactivity in children, assessed as a reduction in respiratory sinus arrhythmia (RSA) in response to a challenge, and better emotional and behavioral regulation (Calkins, Blandon, Williford, & Keane, 2007; Calkins & Keane, 2004; El‐Sheikh, Harger, & Whitson, 2001; Gentzler, Santucci, Kovacs, & Fox, 2009; Graziano & Derefinko, 2013; Graziano, Keane, & Calkins, 2007). A meta‐analysis covering 44 studies of 4,996 children concluded that there was evidence for small effects of vagal reactivity on externalizing and internalizing symptoms; however, the authors also found substantial heterogeneity across studies (Graziano & Derefinko, 2013).

It is possible that estimates of overall effects are attenuated by sex differences. As long as 20 years ago, Eisenberg et al. (1995) reported that higher vagal tone was associated with improved social competence and emotion regulation in boys but with poorer functioning in these areas in girls. More recent studies have also found the same pattern of sex differences (Hinnant & El‐Sheikh, 2013; Morales, Beekman, Blandon, Stifter, & Buss, 2015). For example, Hinnant and El‐Sheikh (2013) found that girls with higher vagal reactivity during a frustration task were more likely to be in a profile characterized by high externalizing and high internalizing symptoms, whereas this was true for boys with weaker vagal reactivity or vagal tone augmentation during the same task. More recently, Morales et al. (2015) found similar results. In this study, exuberance temperament was predictive of externalizing symptoms in girls when these showed heightened vagal reactivity at 24 and 42 months, and in boys only when these showed augmented vagal tone at 24 months.

We have previously shown that low birth weight and prenatal maternal anxiety was associated with increasing vagal reactivity assessed at 29 weeks in females, and with decreasing vagal reactivity in males (Tibu et al., 2014). We now ask whether this sex difference in vagal reactivity is associated with a distinct development of psychiatric symptoms and, in particular, ODD symptoms.

In the last years, there has been increasing evidence for distinct emotional dysregulation and disruptive behavior dimensions in ODD (Burke et al., 2014; Cavanagh et al., 2017; Ezpeleta, Granero, de la Osa, Penelo, & Domenech, 2012; Stringaris & Goodman, 2009b). These two dimensions – named ‘headstrong’ and ‘irritability’ – probably have distinct etiologies (Stringaris, Zavos, Leibenluft, Maughan, & Eley, 2012) and predict different longitudinal outcomes (Stringaris & Goodman, 2009a; Vidal‐Ribas, Brotman, Valdivieso‐Lopez, Leibenluft, & Stringaris, 2016), with irritability associated with depression and headstrong with conduct problems and attention‐deficit hyperactivity disorder (ADHD) (Burke, Hipwell, & Loeber, 2010; Stringaris & Goodman, 2009a). Sex differences in predictions from ODD have also been described; ODD in girls is associated with later depression and anxiety, whereas in boys, it is associated with conduct disorders (Rowe, Costello, Angold, Copeland, & Maughan, 2010).

Given the existence of sex differences in vagal reactivity and phenotype presentation and outcomes of ODD, we examined the following hypotheses. First, in males, *decreased* vagal reactivity will be associated with later ODD symptoms, but in females, elevated ODD symptoms will be associated with *increased* vagal reactivity. Second, in boys, the association will be with the risk for later conduct problems, namely headstrong symptoms, and in girls with the risk for depression, namely irritability.

## Methods

### Participants

The participants were members of the Wirral Child Health and Development Study, a prospective epidemiological longitudinal study of consecutive first‐time mothers who booked for antenatal care at 12 weeks’ gestation between February 12, 2007, and October 29, 2008, with surviving singleton births. Approval for the procedures was obtained from the Cheshire North and West Research Ethics Committee (UK). Potential participants were introduced to the study by research midwives who obtained informed consent.

The study design has been previously described (Sharp, Hill, Hellier, & Pickles, 2015; Sharp et al., 2012; Tibu et al., 2014). Recruited from consecutive attendees at the 20‐week prenatal check, the full cohort of 1,233 primiparous mothers with a mean age of 26.8 years (*SD* = 5.8, range 18–51) were all women who gave birth to a live, singleton baby eligible for longitudinal follow‐up. Obstetric outcomes were gathered from medical records, and the whole cohort completed assessments at 9–12 weeks, 14 months, 3.5 years, and 5 years postnatally. Additional assessments were undertaken on a stratified random subsample of 316 mothers at 32 weeks’ pregnancy, 5 weeks, 29 weeks, and 2.5 years. This design enables intensive measurement to be completed on the stratified subsample, such as that used in this study to assess vagal reactivity, but also allows the collection of other measures across the whole cohort. The stratification variable, interpartner psychological abuse, was chosen for its known association with a variety of risk factors for early child development (Moffitt et al., 1997). All participants scoring above the threshold for psychological abuse toward themselves or their partners at 20 weeks’ gestation were eligible for inclusion in the intensive sample plus a random selection from those below the threshold. Within the intensively assessed stratified subsample, 51% were drawn from the women with high psychosocial risk and 49% from those with low psychosocial risk. The stratifier has proved effective in increasing risk within the high stratum being associated with prenatal risks (Sharp et al., 2015). In this study, the stratifier was associated with ODD symptoms yielding correlations at 2.5, 3.5, and 5 years of 0.18 (*p* = .006), 0.13 (*p* < .001) and 0.19 (*p* < .001), respectively.

A participant flow diagram of this study can be seen in Figure S1 in the supporting information, available online. For this report, whole cohort data (*n* = 1,233) were available for stratification and confounders measures, and *n* = 826 and *n* = 770 for maternal reports of child ODD at 3.5 and 5 years postnatal, and for the stratified subsample *n* = 270 for infant vagal reactivity at 29 weeks and *n* = 253 for maternal reports of child ODD at 2.5 years. Based on *t*‐test and chi‐square results, children with measures on all procedures were not different from the initial intensive sample in terms of mother's age (*p* = .470), sex (*p* = .468), socioeconomic status (*p* = .669), or prenatal maternal depression (*p* = .793).

### Measures

#### Respiratory Sinus Arrhythmia

Respiratory sinus arrhythmia was computed from an ECG recording made during five procedures. These five methods were (a) the helper‐hinderer (Hamlin, Wynn, & Bloom, 2007), (b) the novel toy exploration (Calkins & Dedmon, 2000), and three sequences of the ‘Still‐Face’, (c) face‐to‐face engagement with mother, (d) the still face, and (e) repair (Tronick, Als, Adamson, Wise, & Brazelton, 1978). The patterns of RSA across the five procedures were very similar in males and females, and there were no significant sex differences in any procedure (Tibu et al., 2014). A principal components analysis yielded a single factor with an eigenvalue of 3.54 that explained 70.73% of the total variance (Sharp et al., 2012). All five RSA values loaded highly on to the factor (factor loadings .86, .84, .84, .84, .82) supporting the existence of a latent variable which could be construed as ‘resting’ or ‘baseline’. Post hoc tests in repeated‐measures ANOVA showed that RSA during the Still Face was significantly lower than in each of the other four procedures (all values of *p* < .01) consistent with vagal reactivity (i.e. suppression) to the stressor. Baseline vagal tone was measured by the average RSA across the helper‐hinderer, novel toy, engagement, and repair conditions. Vagal reactivity was measured as this average minus the RSA under the still‐face condition. Therefore, larger values indicated larger RSA reactivity. Details of the procedures and the recording of RSA can be found in Tibu et al. (2014) and Appendix S1 in the online supporting information.

#### Oppositional defiant disorder symptoms

Maternal report of child ODD symptoms was assessed at 2.5, 3.5, and 5 years using the preschool Child Behavior Checklist (CBCL), which has been extensively employed in studies of child and adolescent emotional and behavioral disorders (Achenbach & Rescorla, 2000). ODD dimensions of *irritability* and *headstrong* symptoms were generated following the results of previous confirmatory factor analyses (CFA) in adolescents and adults (Stringaris et al., 2012) across the items of the ODD subscale, with three items in each dimension. Cronbach's alpha coefficients across time ranged 0.60–0.74 for *irritability* and 0.70–0.76 for *headstrong*. A full description of the CFA is in Appendix S2 in the supporting information, available online.

#### Confounders

Previous studies have shown that younger maternal age (Fergusson & Woodward, 1999; Harden et al., 2007), socioeconomic status (Bradley & Corwyn, 2002), and maternal depressive symptoms (Brennan et al., 2000) are associated with emotional and behavioral problems in offspring. We, therefore, employed these measures as covariates in the analyses.

Maternal age was recorded in the 20 weeks’ gestation questionnaire with the extensive sample (*n* = 1,233).

Socioeconomic status was measured using the revised English Index of Multiple Deprivation (IMD); (Noble et al., 2004) based on data collected from the UK Census in 2001. According to this system, postcode areas in England are ranked from most deprived (i.e. IMD of 1) to least deprived (i.e. IMD of 32,482) based on deprivation in seven domains: income, employment, health, education and training, barriers to housing and services, living environment, and crime. All mothers were given IMD ranks according to the postcode of the area where they lived and assigned to a quintile based on the UK distribution of deprivation.

Maternal depression was assessed by self‐report using the Edinburgh Postnatal Depression Scale (Cox, Chapman, Murray, & Jones, 1996) at 2.5 and 3.5 years and the Center for Epidemiologic Studies Depression Scale (Radloff, 1977) at 5 years. Depression scores at these three assessment points were included in analyses to control for possible biasing effects on maternal reports of child ODD symptoms.

### Statistical analyses

The sample design and attrition were accounted for in the estimates of descriptive statistics by the use of inverse probability weighting (Lehtonen & Pahkinen, 2004). Pickles, Dunn, and Vazquez‐Barquero (1995) provide a description specific to this type of study design.

To test our developmental hypotheses, we fitted two structural equation models (*SEM*) estimated using full maximum likelihood (MLR) and the auxiliary command, a method which enables participants with incomplete observations to be included under the assumption of the missingness being missing‐at‐random (MAR) (Graham, 2003). This method yields estimates that account for the sample stratification and for attrition associated with covariates and observed values of outcomes (e.g. RSA under still face or ODD symptoms). The five measures of RSA were incorporated into the model as dependent indicators of vagal tone and vagal reactivity factors, enabling their observation only in the intensive sample to be accounted for under the MAR assumption. For examining sex differences, we made use of the ‘knownclass’ approach for the two groups and Wald tests of main‐effect and interaction contrasts formed as linear functions of parameters defined within model constraints.

In the first model, we examined relationships to overall ODD symptoms and in the second to the ODD dimensions of irritability and headstrong. The standardized estimates reported are from the model in which all observed variables and factors are scaled to have unit variance. Sex difference contrasts were defined in the traditional effect size manner by semi‐standardized effects where the predicted variable had unit variance for both males and females.

## Results

### Descriptive statistics

Table 1 gives summary statistics for the measures for males and females separately, as well as for the whole sample. Mothers reported higher levels of overall ODD symptoms in boys than in girls at 3.5 and 5 years. This difference was accounted for mainly by higher levels of irritability symptoms in boys at ages 3.5 and 5 years. Correlations between the main measures for the whole cohort as well as boys and girls separately are provided in Tables S1–S3, available online in the supporting information.

**Table 1:** Sample characteristics

	Whole sample		Boys				Girls				
	*N*	Mean (*SD*)	*N*	Mean (*SD*)	Min	Max	*N*	Mean (*SD*)	Min	Max	*T*‐test *p*‐value
Mother's age at 20‐week scan (years)	1,233	26.8 (5.8)	599	26.8 (5.8)	18	51	634	26.8 (5.9)	18	43	.862
Socioeconomic status (IMD)	1,230	32.9 (20.2)	598	33.3 (20.8)	1.7	80.6	632	32.6 (19.6)	1.67	79.7	.519
Maternal depression											
2.5 years	242	5.2 (5)	115	4.9 (5.1)	0	24	127	5.5 (4.9)	0	23	.391
3.5 years	812	5 (4.4)	389	5.2 (4.6)	0	24	423	4.9 (4.2)	0	19	.423
5 years	738	7.5 (7.8)	352	8.1 (8.7)	0	55	386	7 (6.9)	0	38	**.046**
RSA											
Helper hinderer	270	3.3 (0.8)	131	3.2 (0.8)	1.1	5.8	139	3.4 (0.9)	1	5.5	.366
Novel toy	266	3 (0.8)	128	2.9 (0.75)	1.5	4.7	138	3 (0.9)	0.4	5.8	.464
Engagement	257	3..3 (0.8)	123	3.4 (0.8)	1.6	5.2	134	3.3 (0.9)	0.6	6	.301
Still face	253	2.8 (0.8)	121	2.8 (0.7)	0.6	4.6	132	2.9 (0.8)	0.7	5	.550
Repair	247	3.3 (1)	119	3.4 (0.9)	1.3	5.8	128	3.3 (1)	0.6	5.9	.421
Vagal reactivity	244	0.5 (0.6)	116	0.5 (0.6)	−0.7	2.1	128	0.4 (0.6)	−0.9	2	.320
ODD symptoms											
2.5 years	245	3.2 (2.2)	123	3.3 (2.2)	0	12	126	3 (2.1)	0	12	.269
3.5 years	808	2.9 (2.5)	395	3.1 (2.5)	0	12	431	2.8 (2.4)	0	12	**.035**
5 years	758	2.7 (2.5)	369	2.9 (2.6)	0	12	401	2.5 (2.2)	0	12	**.017**
ODD irritability											
2.5 years	248	1.6 (1.2)	122	1.6 (1.3)	0	6	130	1.6 (1.2)	0	6	.624
3.5 years	817	1.4 (1.4)	395	1.5 (1.4)	0	6	431	1.3 (1.2)	0	6	**.035**
5 years	763	1.3 (1.4)	369	1.5 (1.5)	0	6	401	1.2 (1.3)	0	6	**.012**
ODD headstrong											
2.5 years	249	1.6 (1.3)	123	1.4 (1.3)	0	6	130	1.4 (1.2)	0	6	.136
3.5 years	816	1.5 (1.4)	395	1.6 (1.4)	0	6	431	1.4 (1.3)	0	6	.090
5 years	762	1.3 (1.4)	369	1.4 (1.4)	0	6	401	1.3 (1.3)	0	6	.063

RSA, respiratory sinus arrhythmia; ODD, oppositional defiant disorder; IMD, index of multiple deprivation. *p* < .05 in bold.

### Confirmatory factor analysis of ODD symptoms

Table S4, available online, shows that compared with the one‐factor model, a two‐factor model that decomposed ODD into irritability and headstrong showed significant improvements. A full report of the results of the CFA is in the supporting information, available online.

### SEM applied to the analysis of RSA predicting ODD symptoms

**Figure 1:**
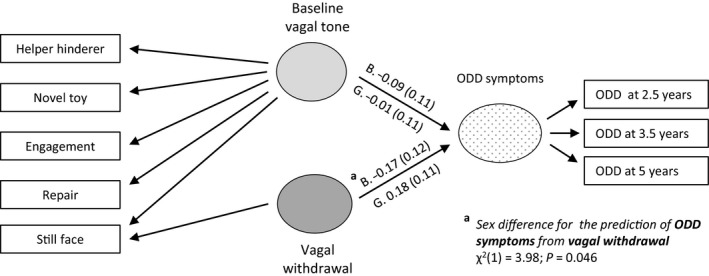
Summary of structural equation modeling analysis of baseline vagal tone and vagal reactivity predicting overall ODD symptoms. Standardized coefficients with standard errors in bold, *p* < .05. G, girls; B, boys

**Figure 2:**
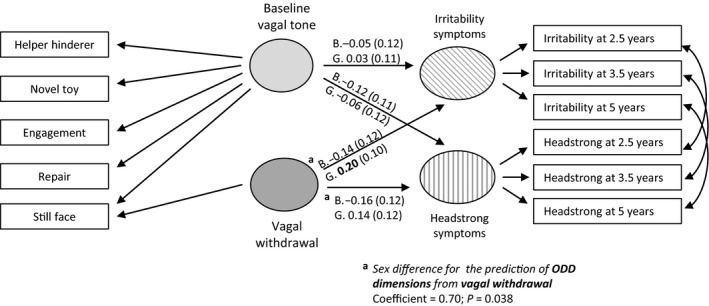
Summary of structural equation modeling analysis of baseline vagal tone and vagal reactivity predicting ODD dimensions of irritability and headstrong symptoms. Standardized coefficients with standard errors in bold, *p* < .05. G, girls; B, boys

The model of interest is shown in Figure 1 for the single ODD symptoms dimension and Figure 2 for the two ODD dimensions, with different parameters being allowed for boys and girls. The RSA measures were decomposed into a general vagal tone factor and vagal reactivity defined by differential response under the Still‐Face condition. Separate effects of vagal tone and vagal reactivity on ODD were then estimated. Though not shown in Figures 1 and 2, to account for the sample stratification, the psychological abuse variable was included as an auxiliary variable freely correlated with all other variables, and the potential confounders of maternal age and socioeconomic status were included as covariates influencing baseline vagal tone, vagal reactivity, and ODD symptoms.

### Vagal tone, vagal reactivity, and ODD symptoms

The model shown in Figure 1 fitted satisfactorily (overall: CFI = .950, RMSEA = .031; males: CFI = .926, RMSEA = .038; females: CFI = .967, RMSEA = .025).

Following our first hypothesis, we tested whether vagal reactivity was differently associated with ODD symptoms in boys and girls. Wald test showed that there was a significant sex difference (*χ* ^2^(1) = 3.98; *p* = .046) arising from the negative association in males (−0.17, 95% CI: −0.40, 0.07, *p* = .159) and positive association in females (0.18, 95% CI: −0.04, 0.40, *p* = .100).

For the association between baseline vagal tone and ODD symptoms, there was no sex difference (*χ* ^2^(1) = 0.45; *p* = .504) as associations were close to zero, both in boys (−0.09, 95% CI: −0.31, 0.13; *p* = .418) and girls (−0.01, 95% CI: −0.22, 0.21; *p* = .947).

### Vagal tone, vagal reactivity, and ODD irritability and headstrong dimensions

**Figure 3:**
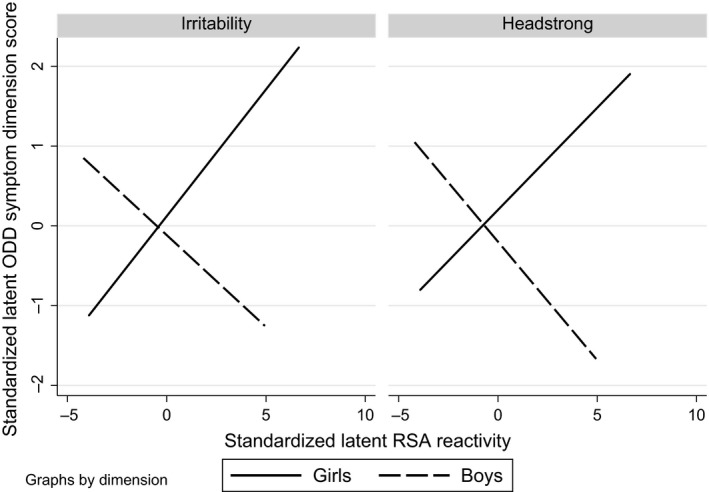
Interplay between sex and respiratory sinus arrhythmia (RSA) reactivity in the prediction of irritability and headstrong ODD symptom dimensions

The model shown in Figure 2 examined dimensional‐specific effects and fitted a little better than the model with the single factor (overall: CFI = .968, RMSEA = .027; males: CFI = .955, RMSEA = .032; females: CFI = .978, RMSEA = .022). For girls, the model estimated a significant positive association of vagal reactivity with irritability (0.20, 95% CI: 0.003, 0.40, *p* = .047) and a smaller nonsignificant association with headstrong (0.14, 95% CI: −0.09, 0.38, *p* = .225). For boys, negative associations were estimated for both dimensions though neither was significant (irritability −0.14, 95% CI: −0.37, 0.10, *p* = .250 and headstrong −0.16, 95% CI: −0.38, 0.07, *p* = .172). These four coefficients were decomposed into an overall average effect, a difference by ODD dimension, a difference by sex, and a ODD dimension by sex interaction. Only the sex difference was significant (coefficient = .70, *p* = .038) there being no evidence for a difference by dimension (coefficient = −.09, *p* = .457) nor any interaction (coefficient = .09, *p* = .701). The pattern of associations is illustrated in Figure 3, where the simple linear fits to the estimated irritability, headstrong, and vagal reactivity factor scores are displayed. The parallel effects on the two symptom dimensions are clear together with a marked difference for boys and girls that is consistent across dimensions (Figure 3).

In the whole sample, vagal reactivity was neither associated with headstrong symptoms (0.01, 95% CI: −0.16, 0.17, *p* = .926) nor with irritability symptoms (0.05, 95% CI: −0.10, 0.20, *p* = .505). Baseline vagal tone was neither associated with headstrong (males: −0.12, 95% CI: −0.32, 0.08, *p* = .255; females: −0.06, 95% CI: −0.28, 0.17, *p* = .630) nor with irritability (males: −0.05, 95% CI: −0.29, 0.18, *p* = .658; females: 0.03, 95% CI: −0.17, 0.24, *p* = .745).

## Discussion

In this study, we first hypothesized that vagal reactivity, assessed as a reduction in RSA to a stressor at 29 weeks, would be associated with ODD symptoms in opposite directions in boys and girls up to age 5 years. The results supported our first hypothesis; there was a significant sex difference due to the opposite directionality in which increasing vagal reactivity was associated with an increase in ODD symptoms in girls and a reduction of ODD symptoms in boys. In contrast, our second hypothesis of dimensional specific effects for each sex received little support.

Our findings are in line with previous studies that have found a Sex by Vagal reactivity interaction in the prediction of future socioemotional outcomes (Hinnant & El‐Sheikh, 2013; Morales et al., 2015). Moreover, the direction of the prediction was the same; that is, higher vagal reactivity was related to poorer outcomes in girls (i.e. more ODD symptoms) and better outcomes in boys (i.e. less ODD symptoms).

We have previously shown that low birth weight and prenatal anxiety were associated with increasing vagal reactivity in females and decreasing vagal reactivity in males (Tibu et al., 2014). In this study, we further showed that these distinct patterns of vagal reactivity are associated with different outcomes. Altogether, these findings expand on our understanding of sex differences in psychopathology. It is well established that some sex differences arise from differential exposure to risks that operate in the same way in males and females (Rutter, Caspi, & Moffitt, 2003), and there is growing evidence for sex dimorphic risks, for example, for depression (Costello, Worthman, Erkanli, & Angold, 2007; Quarini et al., 2016). Here, we add a further possibility; that even when the risks do not differ between males and females the mechanisms may do. That is, our findings are consistent with the possibility that males respond to stressors with reduced, and females with increased, arousal and emotional reactivity, and that each confers vulnerability in different ways. The risk of ODD symptoms in boys might arise from a failure to sufficiently activate inhibitory processes, but in girls might be explained from dysregulation of autonomic responses leading to detrimental effects similar to those reported at high or low levels of vagal reactivity (Marcovitch et al., 2010; Obradovic, 2012). Another but not exclusive possibility is that parents might have different expectations from girls and boys in terms of their coping strategies under stressful conditions. Such expectations might result in differential reinforcement of coping strategies in boys and girls and, consequently, in different parenting styles and child–parent interactions (Chang, Olson, Sameroff, & Sexton, 2011). Eventually, even when boys and girls show the same responses to the same stressors, these child–parent interactions might lead to the development of different emotional and behavioral regulation trajectories later in life (Kopala‐Sibley et al., 2017). Finally, as mentioned at the beginning of this paragraph, some evidence also suggests that differences in fetal programming might impact the response to stress challenges (Tibu et al., 2014). It is then possible that prenatal risk factors are associated with later development of psychiatric symptoms through the mediation role of reactivity to stressful life experiences.

Our second set of analyses (i.e. the prediction of ODD dimensions by vagal reactivity) was a first attempt to explain the ODD differences seen in boys and girls (Trepat & Ezpeleta, 2011) and a very first step to try to explain sex differences in the development of externalizing and internalizing disorders later in life (Angold & Rutter, 1992; Lewinsohn, Hops, Roberts, Seeley, & Andrews, 1993). The results of the CFA should be taken with caution. Although the two‐factor model fitted a little better to the data than the single‐factor model, to our knowledge this is the first study to perform a CFA of ODD dimensions in children as young as 2.5 years old and also the first to employ the preschool version of the CBCL to generate these dimensions. Nevertheless, irritable and headstrong dimensions have been previously found in 3‐year‐old preschoolers employing other instruments (Ezpeleta et al., 2012), and these dimensions were associated with different correlates (Ezpeleta, Granero, de la Osa, Trepat, & Domenech, 2015), confirming that the heterogeneity of ODD is present from early in life.

Although ODD symptoms can be considered multidimensional at these early ages, we did not find that vagal reactivity predicted each ODD dimension differently in boys and girls. Instead, our analyses showed that the RSA by Sex interaction found in the prediction of ODD symptoms could not be conclusively explained by a mechanism of each sex being responsive to just one ODD dimension. While the pattern of coefficients was larger in magnitude for irritability in girls and for headstrong in boys, the stronger pattern was that the sex difference was common across the prediction of both ODD dimensions.

In this study, we found a significant Vagal reactivity by Sex interaction. However, as opposed to other studies (Eisenberg et al., 1995; Hinnant & El‐Sheikh, 2013; Morales et al., 2015), we did not find a baseline Vagal tone by Sex interaction in the prediction of neither ODD symptoms nor ODD dimensions. It should be noted, though, that previous studies have employed single measures of baseline vagal tone. By contrast, in our study, we employed a latent variable approach to create a latent vagal tone measure with the shared variance of the vagal tone measured under five tasks, and then generated the vagal reactivity measure by including a differential item contrast. This approach overcomes the methodological limitation of having task‐specific baselines and the interpretation of vagal reactivity limited to a single task. In addition, the measurement of vagal tone in this study was collected in very young children (i.e. 29 weeks), as opposed to preschool (Morales et al., 2015) and school‐aged children (Eisenberg et al., 1995; Hinnant & El‐Sheikh, 2013) in previous studies. Baseline vagal tone has been shown to be moderately stable from preschool or middle childhood onwards (Calkins & Keane, 2004; El‐Sheikh, 2005) but less so in the first years of life (Bornstein & Suess, 2000), which might also have an impact in the results.

We did not find significant effects in the prediction of ODD symptoms within each sex separately, with the exception of RSA reactivity predicting irritability in girls. However, our principal hypothesis was related to the sex difference, and that the effects would be in opposite directions in boys and girls. The absence of clear cut effects in separate analyses of males and females does suggest the need for caution. These results are very similar to the effects reported previously, where interactions were significant, but separate effects were not, or these were only significant for girls (Hinnant & El‐Sheikh, 2013; Morales et al., 2015). It may simply be that the individual effects are quite modest and so in the smaller male and female subsamples, nonsignificant; or that we have identified an early divergence in mechanisms that become more evident over time and in interaction with later environmental exposures. This needs to be examined in future studies.

One clinical implication of the findings described here, taken together with those from previous studies showing similar sex differences, is that interventions and the study of early markers of risk may need to consider different processes in males and females. A second potential implication is that vagal reactivity in infancy may provide a basis for identifying infants with either very high or very low reactivity as being at risk for later disorders, hence creating a way of stratifying samples for early intervention. This may become possible where further moderators of the effects, such as exposure to adverse environments, can be identified (Obradovic, 2012), enabling combinations of factors to be used.

This study included a number of strengths. First, our findings are based on a population‐based sample followed from pregnancy to age 5 years, with good sample retention. Second, as mentioned earlier, a latent variable approach to the analysis of RSA across contrasting procedures provided strong evidence of an overall level of vagal tone evident in low‐ and high‐stress conditions. The differential item functioning approach provided a strong test of the specificity of vagal reactivity for later symptoms by first accounting for associations with the general vagal tone latent variable and then testing for any additional association specifically with vagal reactivity, as defined in the model by the contrast between vagal tone in the still face and the general latent variable. This approach has provided evidence for associations with vagal reactivity to a social stressor, but not with vagal tone under low‐stress conditions, of prenatal risks (Tibu et al., 2014) and now of subsequent child behaviors. An additional strength of this study was that the ODD dimensions of irritability and headstrong were extracted by CFA, as has been done previously (Stringaris & Goodman, 2009b).

The findings of this study must be also considered in the light of its limitations. Although the design allowed us to examine prospective associations over a period of more than 4 years, that leaves many questions regarding the intervening autonomic, emotional, and behavioral processes yet to be investigated. We cannot assume causality from the reported associations because there may be unmeasured confounders, such as genetic factors and other sociodemographic indicators, affecting these relations. Similarly, we cannot assume that the sex differences arose from the hypothesized sex‐dependent processes outlined earlier. Instead, it is possible that they arose from differences in the ways boys and girls respond to the Still‐Face stressor. For example, different consequences of early social interaction with mothers in males and females have been described, indicating that males may be more affected in the still‐face procedure by maternal sensitivity than girls (Warren & Simmens, 2005; Weinberg, Tronick, Cohn, & Olson, 1999). As a result, maternal sensitivity may be more strongly associated with vagal reactivity in males than in females. A general concern regarding studies reporting statistical interactions is that they run an elevated risk of false positives. This is particularly the case where multiple exploratory analyses of interactions between predictor variables are conducted but less of a concern in studies which, as this study does, examines interactions with sex of infant in the light of the available literature with a hypothesized direction of effect. We sought to minimize the risk further by modeling the outcome as a latent variable requiring contributions from associations with oppositional defiant disorder symptoms across three time points spanning 2.5 years. Another concern is the generalization of findings. Socioeconomic conditions on the Wirral range between the deprived inner‐city and affluent suburbs, but with very low numbers from ethnic minorities. Our analysis correct for the over‐representation of high deprivation in the sample using the deprivation index that reflects the UK population. However, we do not know whether the findings generalize to populations with higher representations of ethnic minorities. Finally, although this is among the largest studies examining the impact of early vagal reactivity, our power to discriminate the detailed differences in developmental processes for boys and girls is limited. In particular, further studies with longer follow‐ups are required to exclude the possibility of boys preferentially responding on the headstrong dimension and girls on the irritability dimension.

## Conclusion

Physiological reactivity to a stressful situation predicts differently ODD symptoms in boys and girls very early in life. This finding might contribute to our understanding of the several mechanisms involved on the later development of distinct psychiatric disorders in boys and girls. In the future, large longitudinal studies should test whether vagal reactivity to stress measured in infancy is predictive of distinct psychiatric disorders in adolescent boys and girls.

**Key points**

- Vagal reactivity to stress plays a role in emotional regulation and the development of psychiatric symptoms, yet results have been inconsistent probably due to different effects in boys and girls.
- In a population‐based sample, vagal reactivity at 29 weeks was associated with increasing ODD symptoms in girls and decreasing ODD symptoms in boys up to 4 years later.
- This sex by vagal reactivity interaction was common for both ODD dimensions, named irritability and headstrong, with no sex by dimension‐specific associations.
- Boys and girls share the same risk factors, but they differ in the mechanisms that make them vulnerable to psychiatric problems later in life.

## Supporting information


**Appendix S1.** Acquisition of respiratory sinus arrhythmia (RSA) and description of procedures employed to compute a general factor of vagal tone.
**Appendix S2.** Confirmatory factor analysis of ODD symptoms.
**Figure S1.** Participant flow diagram.
**Table S1.** Correlations between relevant measures in the whole sample.
**Table S2.** Correlations between relevant measures in boys.
**Table S3.** Correlations between relevant measures in girls.
**Table S4.** Model fit of ODD symptoms in Confirmatory Factor Analyses.
