# Supplementary material for: Sex differences in the associations between vagal reactivity and oppositional defiant disorder symptoms
Source: J Child Psychol Psychiatry. 2017 Jun 2;58(9):988–97. doi: 10.1111/jcpp.12750 (PMC5575540; doi:10.1111/jcpp.12750)
Supplement: Supplementary file 1 — Appendix S1. Acquisition of respiratory sinus arrhythmia (RSA) and description of procedures employed to compute a general factor of vagal tone. Appendix S2. Confirmatory factor analysis of ODD symptoms. Figure S1. Participant flow diagram. Table S1. Correlations between relevant measures in the whole sample. Table S2. Correlations between relevant measures in boys. Table S3. Correlations between relevant measures in girls. Table S4. Model fit of ODD symptoms in Confirmatory Factor Analyses. [file JCPP-58-988-s001.docx]

**Online Supporting Information for - Sex Differences in the Associations Between Vagal Reactivity and Oppositional Defiant Disorder Symptoms - by Vidal-Ribas et al.**


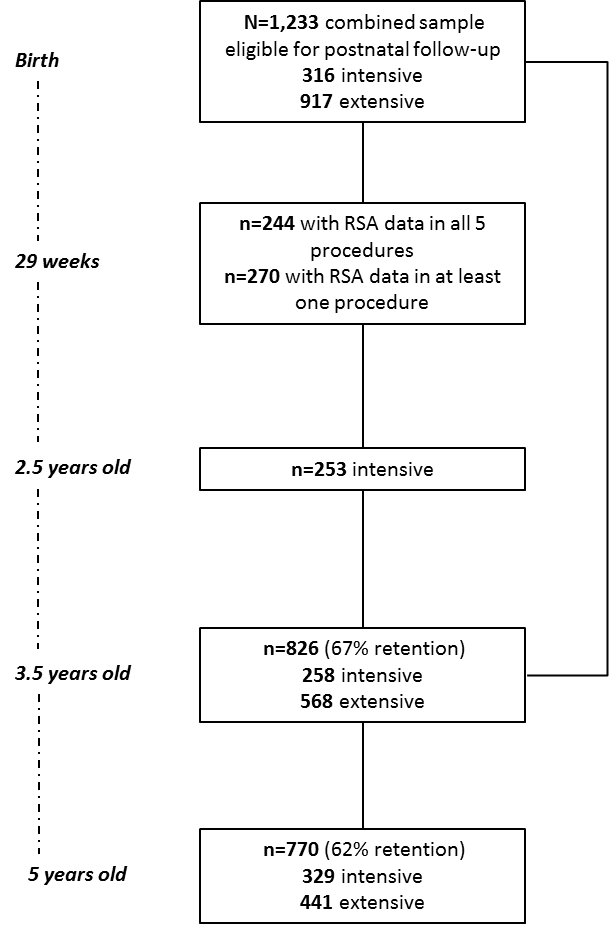


**Fig S1.** Participant flow diagram. *Note*: Although not relevant for this study, there were two assessments points before ‘birth’ and two more assessments points between ‘birth’ and ‘29 weeks’.

**Appendix S1: Acquisition of respiratory sinus arrhythmia (RSA) and description of procedures employed to compute a general factor of vagal tone**

RSA was computed from an electrocardiograph (ECG) recording made during five procedures described below. The recording was made from three Biopac (Biopac Systems, Inc., USA) pediatric disposable ECG electrodes placed on the infant’s back connected to a Biopac Student MP35 acquisition unit box. The cardiac measurements were performed using the 3.9.1 version of the AcqKnowledge data recording software installed on a Windows XP laptop-computer. The output was transmitted to the computer and stored for later off-line extraction of heart period data. All assessments were recorded on DVDs using a split-screen procedure, with three video outputs from the cameras and one showing the ECG trace. An electronic timer was also shown on the screen to code timings for procedures. RSA was calculated by Cardioedit software using a procedure developed by Porges (1985). First, R–R (i.e., interbeat) intervals are timed to the nearest millisecond, which results in a time series of consecutive heart periods (HP). Then an algorithm is applied to the sequential interbeat intervals data that uses a third-order 21-point moving polynomial filter (Porges & Bohrer, 1990) that detrends periodicities in HP slower than RSA. A bandpass filter extracts the variance of HP within the frequency band of spontaneous respiration in infants (0.24–1.04 Hz). Finally, RSA is derived by calculating the natural log of this variance and is reported in units of log normal (ms).

*Procedure 1, The Helper-Hinderer* is an experimental paradigm developed to assess whether infants favour prosocial acts (Hamlin, Wynn, & Bloom, 2007). The infant is seated on the mother’s lap and views a large display (3x5 feet) situated in front of him/her approximately 6 feet away in which a coloured shape (square, circle, triangle) with googly eyes is shown either helping another up a slope (helper trial) or hindering another’s progress up the slope (hinderer trial). Helping trials and hindering trials are alternated throughout and the series of learning trials are ended once the infant has shown a predetermined level of habituation to the stimuli, or when the maximum number of pre-set trials has been reached (14 trials). Once the learning trials have ended the infant are given a preference task, between the helper-shape or hinderer-shape. The duration of the learning procedure is not standard and varies depending on how quickly the infant habituates to the presentation of the stimuli. In the current study the mean duration of the procedure was 3.74 minutes, *SD* 1.20, minimum 0.88 minutes, maximum 8.09 minutes. RSA was calculated for the last 2 minutes of this procedure to ensure standardisation of infants’ looking times.

*Procedure 2 - The Novel Toy Exploration Procedure* is a 2-minute episode in which the infant is presented at a table with a 4-facet triangular pyramid-shaped toy to explore for two minutes while sitting on mother’s knees. This has been used in previous studies to assess baseline vagal tone (Calkins & Dedmon, 2000).

*Procedures 3, 4, and 5 - Still-face procedure* The third, fourth and fifth procedures were conducted with the infant in a high chair facing the mother. They comprised two minutes of face to face playful interactions without toys, followed by two minutes during which the mother was asked to be unresponsive to her child’s communications (the ‘Still Face’), after which she became again responsive (the ‘repair’) (Tronick, Als, Adamson, Wise, & Brazelton, 1978). The Still Face has been used extensively in studies of vagal reactivity (Moore, 2009; Moore & Calkins, 2004)

| **Table S1. Correlations between relevant measures in the whole sample** | | | | | | | | | | | | | | | |
| --- | --- | --- | --- | --- | --- | --- | --- | --- | --- | --- | --- | --- | --- | --- | --- |
|  |  | 1 | 2 | 3 | 4 | 5 | 6 | 7 | 8 | 9 | 10 | 11 | 12 | 13 | 14 |
|  |  | r | r | r | r | r | r | r | r | r | r | r | r | r | r |
|  |  | *p*-value | *p*-value | *p*-value | *p*-value | *p*-value | *p*-value | *p*-value | *p*-value | *p*-value | *p*-value | *p*-value | *p*-value | *p*-value | *p*-value |
|  |  | n | n | n | n | n | n | n | n | n | n | n | n | n | n |
|  |  |  |  |  |  |  |  |  |  |  |  |  |  |  |  |
| 1 | Mother’s age at 20 week scan (years) |  |  |  |  |  |  |  |  |  |  |  |  |  |  |
|  |  |  |  |  |  |  |  |  |  |  |  |  |  |  |  |
|  |  |  |  |  |  |  |  |  |  |  |  |  |  |  |  |
| 2 | Socioeconomic status (IMD) | -.367^***^ |  |  |  |  |  |  |  |  |  |  |  |  |  |
|  |  | .000 |  |  |  |  |  |  |  |  |  |  |  |  |  |
|  |  | 1230 |  |  |  |  |  |  |  |  |  |  |  |  |  |
| 3 | RSA | -.046 | .041 |  |  |  |  |  |  |  |  |  |  |  |  |
|  |  | .473 | .527 |  |  |  |  |  |  |  |  |  |  |  |  |
|  |  | 244 | 244 |  |  |  |  |  |  |  |  |  |  |  |  |
| 4 | Maternal depression 2.5 years | -.024 | .119 | -.019 |  |  |  |  |  |  |  |  |  |  |  |
|  |  | .713 | .064 | .781 |  |  |  |  |  |  |  |  |  |  |  |
|  |  | 242 | 242 | 207 |  |  |  |  |  |  |  |  |  |  |  |
| 5 | Maternal depression 3.5 years | -.109^**^ | .101^**^ | .045 | .584^***^ |  |  |  |  |  |  |  |  |  |  |
|  |  | .002 | .004 | .508 | .000 |  |  |  |  |  |  |  |  |  |  |
|  |  | 812 | 810 | 218 | 227 |  |  |  |  |  |  |  |  |  |  |
| 6 | Maternal depression 5 years | -.129^***^ | .135^***^ | -.009 | .472^***^ | .509^***^ |  |  |  |  |  |  |  |  |  |
|  |  | .000 | .000 | .903 | .000 | .000 |  |  |  |  |  |  |  |  |  |
|  |  | 738 | 735 | 201 | 215 | 682 |  |  |  |  |  |  |  |  |  |
| 7 | ODD symptoms 2.5 years | -.107 | .039 | .003 | .231^***^ | .110 | .148^*^ |  |  |  |  |  |  |  |  |
|  |  | .094 | .544 | .971 | .000 | .097 | .029 |  |  |  |  |  |  |  |  |
|  |  | 245 | 245 | 208 | 234 | 230 | 218 |  |  |  |  |  |  |  |  |
| 8 | ODD symptoms 3.5 years | -.114^**^ | .103^**^ | .005 | .243^***^ | .212^***^ | .213^***^ | .612^***^ |  |  |  |  |  |  |  |
|  |  | .001 | .004 | .943 | .000 | .000 | .000 | .000 |  |  |  |  |  |  |  |
|  |  | 808 | 806 | 216 | 225 | 785 | 683 | 228 |  |  |  |  |  |  |  |
| 9 | ODD symptoms 5 years | -.083^*^ | .158^***^ | .065 | .303^***^ | .216^***^ | .225^***^ | .492^***^ | .646^***^ |  |  |  |  |  |  |
|  |  | .022 | .000 | .343 | .000 | .000 | .000 | .000 | .000 |  |  |  |  |  |  |
|  |  | 758 | 756 | 216 | 228 | 706 | 725 | 230 | 704 |  |  |  |  |  |  |
| 10 | Irritability 2.5 years | -.197^**^ | .099 | .027 | .302^***^ | .174^**^ | .190^**^ | .860^***^ | .556^***^ | .425^***^ |  |  |  |  |  |
|  |  | .002 | .122 | .693 | .000 | .008 | .005 | .000 | .000 | .000 |  |  |  |  |  |
|  |  | 248 | 248 | 210 | 237 | 233 | 221 | 245 | 230 | 233 |  |  |  |  |  |
| 11 | Irritability 3.5 years | -.171^***^ | .158^***^ | .016 | .227^**^ | .209^***^ | .202^***^ | .492^***^ | .902^***^ | .584^***^ | .555^***^ |  |  |  |  |
|  |  | .000 | .000 | .815 | .001 | .000 | .000 | .000 | .000 | .000 | .000 |  |  |  |  |
|  |  | 817 | 815 | 217 | 227 | 793 | 691 | 229 | 808 | 711 | 232 |  |  |  |  |
| 12 | Irritability 5 years | -.138^***^ | .192^***^ | .074 | .299^***^ | .218^***^ | .213^***^ | .467^***^ | .606^***^ | .916^***^ | .458^***^ | .605^***^ |  |  |  |
|  |  | .000 | .000 | .281 | .000 | .000 | .000 | .000 | .000 | .000 | .000 | .000 |  |  |  |
|  |  | 763 | 761 | 217 | 229 | 710 | 729 | 231 | 709 | 758 | 234 | 716 |  |  |  |
| 13 | Headstrong 2.5 years | .021 | -.041 | -.027 | .104 | .031 | .083 | .862^***^ | .498^***^ | .428^***^ | .483^***^ | .288^***^ | .348^***^ |  |  |
|  |  | .747 | .515 | .699 | .108 | .640 | .218 | .000 | .000 | .000 | .000 | .000 | .000 |  |  |
|  |  | 249 | 249 | 212 | 238 | 234 | 220 | 245 | 232 | 234 | 245 | 233 | 235 |  |  |
| 14 | Headstrong 3.5 years | -.033 | .030 | -.005 | .198^**^ | .169^***^ | .171^***^ | .592^***^ | .899^***^ | .581^***^ | .431^***^ | .622^***^ | .489^***^ | .595^***^ |  |
|  |  | .347 | .392 | .947 | .003 | .000 | .000 | .000 | .000 | .000 | .000 | .000 | .000 | .000 |  |
|  |  | 816 | 814 | 218 | 227 | 793 | 687 | 230 | 808 | 709 | 232 | 808 | 714 | 234 |  |
| 15 | Headstrong 5 years | -.016 | .095^**^ | .046 | .251^***^ | .170^***^ | .198^***^ | .427^***^ | .581^***^ | .914^***^ | .316^***^ | .468^***^ | .675^***^ | .428^***^ | .576^***^ |
|  |  | .658 | .009 | .497 | .000 | .000 | .000 | .000 | .000 | .000 | .000 | .000 | .000 | .000 | .000 |
|  |  | 762 | 760 | 217 | 229 | 708 | 729 | 231 | 706 | 758 | 234 | 714 | 758 | 235 | 711 |
| *. Correlation is significant at the 0.05 level (2-tailed); **. Correlation is significant at the 0.01 level (2-tailed); ***. Correlation is significant at the 0.001 level (2-tailed). | | | | | | | | | | | | | | | |

| **Table S2. Correlations between relevant measures in boys** | | | | | | | | | | | | | | | |
| --- | --- | --- | --- | --- | --- | --- | --- | --- | --- | --- | --- | --- | --- | --- | --- |
|  |  | 1 | 2 | 3 | 4 | 5 | 6 | 7 | 8 | 9 | 10 | 11 | 12 | 13 | 14 |
|  |  | r | r | r | r | r | r | r | r | r | r | r | r | r | r |
|  |  | *p*-value | *p*-value | *p*-value | *p*-value | *p*-value | *p*-value | *p*-value | *p*-value | *p*-value | *p*-value | *p*-value | *p*-value | *p*-value | *p*-value |
|  |  | n | n | n | n | n | n | n | n | n | n | n | n | n | n |
|  |  |  |  |  |  |  |  |  |  |  |  |  |  |  |  |
| 1 | Mother’s age at 20 week scan (years) |  |  |  |  |  |  |  |  |  |  |  |  |  |  |
|  |  |  |  |  |  |  |  |  |  |  |  |  |  |  |  |
|  |  |  |  |  |  |  |  |  |  |  |  |  |  |  |  |
| 2 | Socioeconomic status (IMD) | -.339*** |  |  |  |  |  |  |  |  |  |  |  |  |  |
|  |  | .000 |  |  |  |  |  |  |  |  |  |  |  |  |  |
|  |  | 598 |  |  |  |  |  |  |  |  |  |  |  |  |  |
| 3 | RSA | -.026 | .152 |  |  |  |  |  |  |  |  |  |  |  |  |
|  |  | .783 | .103 |  |  |  |  |  |  |  |  |  |  |  |  |
|  |  | 116 | 116 |  |  |  |  |  |  |  |  |  |  |  |  |
| 4 | Maternal depression 2.5 years | -.029 | .158 | -.134 |  |  |  |  |  |  |  |  |  |  |  |
|  |  | .754 | .091 | .194 |  |  |  |  |  |  |  |  |  |  |  |
|  |  | 115 | 115 | 96 |  |  |  |  |  |  |  |  |  |  |  |
| 5 | Maternal depression 3.5 years | -.155** | .125* | -.040 | .638*** |  |  |  |  |  |  |  |  |  |  |
|  |  | .002 | .014 | .696 | .000 |  |  |  |  |  |  |  |  |  |  |
|  |  | 389 | 388 | 99 | 107 |  |  |  |  |  |  |  |  |  |  |
| 6 | Maternal depression 5 years | -.134* | .140** | -.072 | .412*** | .484*** |  |  |  |  |  |  |  |  |  |
|  |  | .012 | .009 | .493 | .000 | .000 |  |  |  |  |  |  |  |  |  |
|  |  | 352 | 351 | 94 | 104 | 326 |  |  |  |  |  |  |  |  |  |
| 7 | ODD symptoms 2.5 years | -.139 | .155 | -.161 | .278** | .131 | .107 |  |  |  |  |  |  |  |  |
|  |  | .133 | .092 | .114 | .003 | .170 | .275 |  |  |  |  |  |  |  |  |
|  |  | 119 | 119 | 97 | 111 | 111 | 107 |  |  |  |  |  |  |  |  |
| 8 | ODD symptoms 3.5 years | -.146** | .109* | -.172 | .310** | .224*** | .246*** | .705*** |  |  |  |  |  |  |  |
|  |  | .004 | .031 | .089 | .001 | .000 | .000 | .000 |  |  |  |  |  |  |  |
|  |  | 390 | 389 | 99 | 107 | 381 | 329 | 112 |  |  |  |  |  |  |  |
| 9 | ODD symptoms 5 years | -.094 | .160** | -.146 | .425*** | .206*** | .213*** | .570*** | .686*** |  |  |  |  |  |  |
|  |  | .073 | .002 | .144 | .000 | .000 | .000 | .000 | .000 |  |  |  |  |  |  |
|  |  | 363 | 362 | 101 | 109 | 340 | 347 | 113 | 342 |  |  |  |  |  |  |
| 10 | Irritability 2.5 years | -.211* | .169 | -.142 | .343*** | .241** | .211* | .853*** | .688*** | .524*** |  |  |  |  |  |
|  |  | .020 | .064 | .162 | .000 | .010 | .028 | .000 | .000 | .000 |  |  |  |  |  |
|  |  | 121 | 121 | 99 | 113 | 113 | 109 | 119 | 113 | 115 |  |  |  |  |  |
| 11 | Irritability 3.5 years | -.203*** | .157** | -.124 | .313** | .212*** | .200*** | .539*** | .904*** | .602*** | .670*** |  |  |  |  |
|  |  | .000 | .002 | .220 | .001 | .000 | .000 | .000 | .000 | .000 | .000 |  |  |  |  |
|  |  | 394 | 393 | 100 | 108 | 384 | 332 | 112 | 390 | 344 | 114 |  |  |  |  |
| 12 | Irritability 5 years | -.139** | .193*** | -.140 | .423*** | .219*** | .198*** | .520*** | .643*** | .919*** | .539*** | .620*** |  |  |  |
|  |  | .008 | .000 | .163 | .000 | .000 | .000 | .000 | .000 | .000 | .000 | .000 |  |  |  |
|  |  | 365 | 364 | 101 | 109 | 341 | 348 | 113 | 344 | 363 | 115 | 346 |  |  |  |
| 13 | Headstrong 2.5 years | -.020 | .076 | -.136 | .145 | -.005 | -.011 | .865*** | .527*** | .464*** | .476*** | .267** | .364*** |  |  |
|  |  | .832 | .412 | .182 | .126 | .955 | .913 | .000 | .000 | .000 | .000 | .004 | .000 |  |  |
|  |  | 120 | 120 | 98 | 112 | 112 | 108 | 119 | 113 | 114 | 119 | 113 | 114 |  |  |
| 14 | Headstrong 3.5 years | -.060 | .038 | -.168 | .231* | .193*** | .237*** | .691*** | .893*** | .633*** | .530*** | .614*** | .535*** | .657*** |  |
|  |  | .240 | .456 | .097 | .017 | .000 | .000 | .000 | .000 | .000 | .000 | .000 | .000 | .000 |  |
|  |  | 391 | 390 | 99 | 107 | 382 | 330 | 112 | 390 | 343 | 113 | 390 | 345 | 113 |  |
| 15 | Headstrong 5 years | -.042 | .103 | -.123 | .341*** | .144** | .190*** | .507*** | .616*** | .910*** | .410*** | .482*** | .672*** | .467*** | .624*** |
|  |  | .428 | .050 | .217 | .000 | .008 | .000 | .000 | .000 | .000 | .000 | .000 | .000 | .000 | .000 |
|  |  | 366 | 365 | 102 | 110 | 341 | 350 | 114 | 343 | 363 | 116 | 346 | 363 | 115 | 344 |
| *. Correlation is significant at the 0.05 level (2-tailed); **. Correlation is significant at the 0.01 level (2-tailed); ***. Correlation is significant at the 0.001 level (2-tailed). | | | | | | | | | | | | | | | |

| **Table S3. Correlations between relevant measures in girls** | | | | | | | | | | | | | | | |
| --- | --- | --- | --- | --- | --- | --- | --- | --- | --- | --- | --- | --- | --- | --- | --- |
|  |  | 1 | 2 | 3 | 4 | 5 | 6 | 7 | 8 | 9 | 10 | 11 | 12 | 13 | 14 |
|  |  | r | r | r | r | r | r | r | r | r | r | r | r | r | r |
|  |  | *p*-value | *p*-value | *p*-value | *p*-value | *p*-value | *p*-value | *p*-value | *p*-value | *p*-value | *p*-value | *p*-value | *p*-value | *p*-value | *p*-value |
|  |  | n | n | n | n | n | n | n | n | n | n | n | n | n | n |
|  |  |  |  |  |  |  |  |  |  |  |  |  |  |  |  |
| 1 | Mother’s age at 20 week scan (years) |  |  |  |  |  |  |  |  |  |  |  |  |  |  |
|  |  |  |  |  |  |  |  |  |  |  |  |  |  |  |  |
|  |  |  |  |  |  |  |  |  |  |  |  |  |  |  |  |
| 2 | Socioeconomic status (IMD) | -.395*** |  |  |  |  |  |  |  |  |  |  |  |  |  |
|  |  | .000 |  |  |  |  |  |  |  |  |  |  |  |  |  |
|  |  | 632 |  |  |  |  |  |  |  |  |  |  |  |  |  |
| 3 | RSA | -.070 | -.067 |  |  |  |  |  |  |  |  |  |  |  |  |
|  |  | .429 | .452 |  |  |  |  |  |  |  |  |  |  |  |  |
|  |  | 128 | 128 |  |  |  |  |  |  |  |  |  |  |  |  |
| 4 | Maternal depression 2.5 years | -.013 | .077 | .092 |  |  |  |  |  |  |  |  |  |  |  |
|  |  | .887 | .388 | .338 |  |  |  |  |  |  |  |  |  |  |  |
|  |  | 127 | 127 | 111 |  |  |  |  |  |  |  |  |  |  |  |
| 5 | Maternal depression 3.5 years | -.062 | .075 | .134 | .529*** |  |  |  |  |  |  |  |  |  |  |
|  |  | .203 | .123 | .148 | .000 |  |  |  |  |  |  |  |  |  |  |
|  |  | 423 | 422 | 119 | 120 |  |  |  |  |  |  |  |  |  |  |
| 6 | Maternal depression 5 years | -.124* | .128* | .052 | .562*** | .543*** |  |  |  |  |  |  |  |  |  |
|  |  | .015 | .012 | .595 | .000 | .000 |  |  |  |  |  |  |  |  |  |
|  |  | 386 | 384 | 107 | 111 | 356 |  |  |  |  |  |  |  |  |  |
| 7 | ODD symptoms 2.5 years | -.084 | -.067 | .132 | .190* | .087 | .186 |  |  |  |  |  |  |  |  |
|  |  | .352 | .455 | .166 | .036 | .347 | .050 |  |  |  |  |  |  |  |  |
|  |  | 126 | 126 | 111 | 123 | 119 | 111 |  |  |  |  |  |  |  |  |
| 8 | ODD symptoms 3.5 years | -.083 | .096* | .145 | .188* | .194*** | .161** | .522*** |  |  |  |  |  |  |  |
|  |  | .088 | .049 | .120 | .042 | .000 | .002 | .000 |  |  |  |  |  |  |  |
|  |  | 418 | 417 | 117 | 118 | 404 | 354 | 116 |  |  |  |  |  |  |  |
| 9 | ODD symptoms 5 years | -.069 | .154** | .242** | .202* | .226*** | .232*** | .403*** | .599*** |  |  |  |  |  |  |
|  |  | .169 | .002 | .009 | .028 | .000 | .000 | .000 | .000 |  |  |  |  |  |  |
|  |  | 395 | 394 | 115 | 119 | 366 | 378 | 117 | 362 |  |  |  |  |  |  |
| 10 | Irritability 2.5 years | -.186* | .034 | .163 | .263** | .101 | .166 | .870*** | .432*** | .324*** |  |  |  |  |  |
|  |  | .036 | .705 | .088 | .003 | .272 | .080 | .000 | .000 | .000 |  |  |  |  |  |
|  |  | 127 | 127 | 111 | 124 | 120 | 112 | 126 | 117 | 118 |  |  |  |  |  |
| 11 | Irritability 3.5 years | -.140** | .162** | .136 | .146 | .202*** | .196*** | .458*** | .900*** | .560*** | .448*** |  |  |  |  |
|  |  | .004 | .001 | .143 | .114 | .000 | .000 | .000 | .000 | .000 | .000 |  |  |  |  |
|  |  | 423 | 422 | 117 | 119 | 409 | 359 | 117 | 418 | 367 | 118 |  |  |  |  |
| 12 | Irritability 5 years | -.136** | .190*** | .257** | .195* | .215*** | .223*** | .403*** | .559*** | .913*** | .374*** | .580*** |  |  |  |
|  |  | .007 | .000 | .005 | .033 | .000 | .000 | .000 | .000 | .000 | .000 | .000 |  |  |  |
|  |  | 398 | 397 | 116 | 120 | 369 | 381 | 118 | 365 | 395 | 119 | 370 |  |  |  |
| 13 | Headstrong 2.5 years | .057 | -.154 | .061 | .078 | .076 | .191* | .857*** | .472*** | .380*** | .491*** | .325*** | .321*** |  |  |
|  |  | .523 | .082 | .518 | .382 | .403 | .044 | .000 | .000 | .000 | .000 | .000 | .000 |  |  |
|  |  | 129 | 129 | 114 | 126 | 122 | 112 | 126 | 119 | 120 | 126 | 120 | 121 |  |  |
| 14 | Headstrong 3.5 years | -.009 | .022 | .121 | .179 | .142** | .085 | .489*** | .905*** | .524*** | .337*** | .628*** | .435*** | .526*** |  |
|  |  | .857 | .645 | .190 | .050 | .004 | .109 | .000 | .000 | .000 | .000 | .000 | .000 | .000 |  |
|  |  | 425 | 424 | 119 | 120 | 411 | 357 | 118 | 418 | 366 | 119 | 418 | 369 | 121 |  |
| 15 | Headstrong 5 years | .011 | .086 | .185* | .180 | .196*** | .201*** | .337*** | .540*** | .918*** | .220* | .449*** | .675*** | .381*** | .524*** |
|  |  | .830 | .087 | .047 | .051 | .000 | .000 | .000 | .000 | .000 | .017 | .000 | .000 | .000 | .000 |
|  |  | 396 | 395 | 115 | 119 | 367 | 379 | 117 | 363 | 395 | 118 | 368 | 395 | 120 | 367 |
| *. Correlation is significant at the 0.05 level (2-tailed); **. Correlation is significant at the 0.01 level (2-tailed); ***. Correlation is significant at the 0.001 level (2-tailed). | | | | | | | | | | | | | | | |

**Appendix S2: Confirmatory factor analysis of ODD symptoms**

ODD dimensions of *irritability* and *headstrong* symptoms were generated following the results of previous confirmatory factor analyses (CFA) in adolescents and adults (Stringaris, Zavos, Leibenluft, Maughan, & Eley, 2012) across the items of the ODD subscale. The items employed by Stringaris et al.(Stringaris et al., 2012) were drawn from the Youth Self-Report (ages 11-18) and Adult Self-Report (ages 18-59) versions of the ASEBA family of instruments. These included “have a hot temper”; “stubborn” (for adolescents) or “stubborn, sullen or irritable” (for adults), and “mood/feelings change suddenly” for *irritability*; and “disobey parents” (only for adolescents), “mean to others,” “destroy others’ things,” “disobey at school,” and “tease others a lot” for *headstrong.* In the present study we use the Preschool version of the CBCL and, as some items differ, we provide here the results of CFA from our own study. Based on the CFA from Stringaris et al. (Stringaris et al., 2012) *Irritability* comprised the items “Angry moods“, “Stubborn, sullen or irritable“, and “Temper tantrums or hot temper“; *Headstrong* comprised the items “Defiant“, “Disobedient“, and “Uncooperative“. Each item is scored using a 3-point Likert scale (0-Not true, 1-Somewhat or sometimes true, and 2-Very true or often true).

We used multivariate probit confirmatory factor analysis for ordinal data in Mplus using the WLSMV estimator. To consider a model as showing ‘acceptable’ fit, we required a Confirmatory Fit Index (CFI)>0.90, and Root Mean Square Error Approximation (RMSEA) <0.08, and for a ‘good’ fit, we required a CFI>0.95, and RMSEA<0.06 (Brown, 2006). The χ^2^ difference test was used to compare nested models (i.e. one single factor models against a two-factor model), where improvements in model fit by the nested –less constrained and more parsimonious– model are tested .

**Table S4** shows that although a one-factor model for the ODD items generally gave a satisfactory CFI across all three ages and both sexes, the RMSEA were less so. Chi-square difference tests comparing these one factor models to two-factor models that decomposed ODD into irritability and headstrong showed significant improvements in all cases, with the two-factor models having CFI all >0.95 and RMSEA all less than .07, except among the youngest girls. Correlation between the two factors across the 3 points of assessment ranged 0.70-0.86 in boys and 0.75-0.92 in girls, in both cases the correlation increasing with age. These correlations are in line with previous studies (Ezpeleta, Granero, de la Osa, Penelo, & Domenech, 2012; Stringaris & Goodman, 2009).

| **Table S4*.* Model fit of ODD symptoms in Confirmatory Factor Analyses** | | | | | | | | |
| --- | --- | --- | --- | --- | --- | --- | --- | --- |
|  |  | One –Factor | | Two–Factor | |  |  |  |
|  | n | CFI | RMSEA | CFI | RMSEA | Diff χ^2^ | Diff df | p-value |
| *Boys* |  |  |  |  |  |  |  |  |
| 2.5 years | 123 | 0.907 | 0.187 | 0.957 | 0.138 | 12.14 | 1 | 0.0005 |
| 3.5 years | 395 | 0.971 | 0.105 | 0.992 | 0.058 | 22.30 | 1 | <0.0001 |
| 5 years | 369 | 0.976 | 0.125 | 0.994 | 0.065 | 23.55 | 1 | <0.0001 |
| *Girls* |  |  |  |  |  |  |  |  |
| 2.5 years | 130 | 0.943 | 0.131 | 0.971 | 0.093 | 10.20 | 1 | 0.0014 |
| 3.5 years | 431 | 0.980 | 0.091 | 0.997 | 0.040 | 21.48 | 1 | <0.0001 |
| 5 years | 401 | 0.981 | 0.076 | 0.986 | 0.071 | 4.81 | 1 | 0.0284 |
|  | | | | | | | | |

**References**

Calkins, S. D., & Dedmon, S. E. (2000). Physiological and behavioral regulation in two-year-old children with aggressive/destructive behavior problems. *Journal of abnormal child psychology, 28*, 103-118.

Ezpeleta, L., Granero, R., de la Osa, N., Penelo, E., & Domenech, J. M. (2012). Dimensions of oppositional defiant disorder in 3-year-old preschoolers. *Journal of child psychology and psychiatry, and allied disciplines, 53*, 1128-1138.

Hamlin, J. K., Wynn, K., & Bloom, P. (2007). Social evaluation by preverbal infants. *Nature, 450*, 557-559.

Moore, G. A. (2009). Infants' and mothers' vagal reactivity in response to anger. *Journal of child psychology and psychiatry, and allied disciplines, 50*, 1392-1400.

Moore, G. A., & Calkins, S. D. (2004). Infants' vagal regulation in the still-face paradigm is related to dyadic coordination of mother-infant interaction. *Developmental psychology, 40*, 1068-1080.

Porges, S. W. (1985). *US Patent No. 4,510,944*. Washington, DC: US Patent and Trademark Office.

Porges, S. W., & Bohrer, R. E. (1990). Analyses of periodic processes in psychophysiological research. In J. T. C. L. G. Tassinary (Ed.), *Principles of psychophysiology: Physical, social, and inferential elements* (pp. 708–753). New York: Cambridge University Press.

Stringaris, A., & Goodman, R. (2009). Three dimensions of oppositionality in youth. *Journal of child psychology and psychiatry, and allied disciplines, 50*, 216-223.

Stringaris, A., Zavos, H., Leibenluft, E., Maughan, B., & Eley, T. C. (2012). Adolescent irritability: phenotypic associations and genetic links with depressed mood. *The American journal of psychiatry, 169*, 47-54.

Tronick, E., Als, H., Adamson, L., Wise, S., & Brazelton, T. B. (1978). The infant's response to entrapment between contradictory messages in face-to-face interaction. *Journal of the American Academy of Child Psychiatry, 17*, 1-13.
